# Supplementary material for: Modeling glioblastoma heterogeneity as a dynamic network of cell states
Source: Mol Syst Biol. 2021 Sep 16;17(9):e10105. doi: 10.15252/msb.202010105 (PMC8444284; doi:10.15252/msb.202010105)
Supplement: Supplementary file 5 — Source Data for Figure 3 [file MSB-17-e10105-s001.zip › Figure3A_sourcedata/GSEA_3065/hallmarks_state4.GseaPreranked.1623416296068/HALLMARK_TGF_BETA_SIGNALING.html]

Details for gene set HALLMARK\_TGF\_BETA\_SIGNALING[GSEA]

|  || Dataset | state4 |
| Phenotype | NoPhenotypeAvailable |
| Upregulated in class | na\_pos |
| GeneSet | HALLMARK\_TGF\_BETA\_SIGNALING |
| Enrichment Score (ES) | 0.49343657 |
| Normalized Enrichment Score (NES) | 1.7275515 |
| Nominal p-value | 0.003003003 |
| FDR q-value | 0.020468373 |
| FWER p-Value | 0.045 |
Table: GSEA Results Summary

  

Fig 1: Enrichment plot: HALLMARK\_TGF\_BETA\_SIGNALING      
 Profile of the Running ES Score & Positions of GeneSet Members on the Rank Ordered List

  

| PROBE | GENE SYMBOL | GENE\_TITLE | RANK IN GENE LIST | RANK METRIC SCORE | RUNNING ES | CORE ENRICHMENT || 1 | ID3 |  |  | 2 | 0.730 | 0.1834 | Yes |
| 2 | RAB31 |  |  | 21 | 0.388 | 0.2782 | Yes |
| 3 | ID2 |  |  | 56 | 0.292 | 0.3464 | Yes |
| 4 | ID1 |  |  | 84 | 0.261 | 0.4078 | Yes |
| 5 | FKBP1A |  |  | 365 | 0.161 | 0.4033 | Yes |
| 6 | WWTR1 |  |  | 370 | 0.159 | 0.4428 | Yes |
| 7 | JUNB |  |  | 490 | 0.139 | 0.4587 | Yes |
| 8 | RHOA |  |  | 492 | 0.139 | 0.4934 | Yes |
| 9 | HIPK2 |  |  | 909 | 0.095 | 0.4505 | No |
| 10 | APC |  |  | 1029 | 0.086 | 0.4531 | No |
| 11 | CDKN1C |  |  | 1328 | 0.070 | 0.4227 | No |
| 12 | FNTA |  |  | 1574 | 0.059 | 0.3983 | No |
| 13 | TGIF1 |  |  | 1699 | 0.055 | 0.3923 | No |
| 14 | SMAD1 |  |  | 1784 | 0.053 | 0.3920 | No |
| 15 | SKIL |  |  | 2374 | 0.038 | 0.3069 | No |
| 16 | BMPR2 |  |  | 2440 | 0.036 | 0.3056 | No |
| 17 | SKI |  |  | 2451 | 0.036 | 0.3131 | No |
| 18 | MAP3K7 |  |  | 2540 | 0.034 | 0.3075 | No |
| 19 | CDK9 |  |  | 2603 | 0.033 | 0.3058 | No |
| 20 | TRIM33 |  |  | 2850 | 0.027 | 0.2732 | No |
| 21 | PPM1A |  |  | 2934 | 0.026 | 0.2664 | No |
| 22 | UBE2D3 |  |  | 3057 | 0.024 | 0.2528 | No |
| 23 | FURIN |  |  | 3088 | 0.023 | 0.2539 | No |
| 24 | IFNGR2 |  |  | 3147 | 0.022 | 0.2502 | No |
| 25 | SPTBN1 |  |  | 3155 | 0.022 | 0.2546 | No |
| 26 | XIAP |  |  | 3272 | 0.019 | 0.2408 | No |
| 27 | TGFB1 |  |  | 3283 | 0.019 | 0.2439 | No |
| 28 | CTNNB1 |  |  | 3329 | 0.017 | 0.2410 | No |
| 29 | PPP1R15A |  |  | 3474 | 0.013 | 0.2212 | No |
| 30 | TJP1 |  |  | 3531 | 0.011 | 0.2150 | No |
| 31 | ARID4B |  |  | 3613 | 0.008 | 0.2041 | No |
| 32 | BMP2 |  |  | 4398 | -0.036 | 0.0872 | No |
| 33 | PPP1CA |  |  | 4485 | -0.043 | 0.0843 | No |
| 34 | THBS1 |  |  | 4747 | -0.063 | 0.0583 | No |
| 35 | SERPINE1 |  |  | 5812 | -0.186 | -0.0657 | No |
| 36 | PMEPA1 |  |  | 6009 | -0.241 | -0.0365 | No |
| 37 | SLC20A1 |  |  | 6112 | -0.306 | 0.0242 | No |
Table: GSEA details [plain text format]

  

Fig 2: HALLMARK\_TGF\_BETA\_SIGNALING: Random ES distribution      
 Gene set null distribution of ES for **HALLMARK\_TGF\_BETA\_SIGNALING**
